# Temporal trends in vaccination and antibiotic use among young children in the United States, 2000–2019

**DOI:** 10.1017/ash.2025.10044

**Published:** 2025-07-11

**Authors:** Amanda L. Eiden, Qing Liu, Yoonyoung Choi, Yan Song, Gary S. Marshall, Nicolae Done, Travis Wang, Goran Bencina, James Signorovitch

**Affiliations:** 1 Merck & Co. Inc., Rahway, NJ, USA; 2 Analysis Group Inc., Boston, MA, USA; 3 Norton Children’s and the University of Louisville School of Medicine, Louisville, KY, USA; 4 MSD Spain, Madrid, Spain

## Abstract

**Objective:**

Routine immunization programs may reduce antibiotic use, but few studies have comprehensively examined their impact on antibiotic utilization. We aimed to explore temporal trends in vaccination and antibiotic use among young children in the United States.

**Design:**

Ecological study using the Merative® MarketScan Commercial Claims and Encounters database.

**Methods:**

We analyzed claims data on pediatric vaccine uptake (pneumococcal conjugate, *Haemophilus influenzae* type b, diphtheria-tetanus-pertussis, and influenza) and antibiotic prescriptions and antibiotic-treated respiratory tract infections among US children <5 years during 2000–2019. Vaccination status was assessed annually, and children were categorized based on receipt of all four vaccines, 1–3 vaccines, or no vaccines. Antibiotic prescriptions were classified by spectrum and drug class. Respiratory infections included otitis media, pharyngitis, pneumonia, sinusitis, and viral infections.

**Results:**

Among 6.7 million children, vaccine uptake increased from 32.5% receiving all four vaccines in 2004 to 66.8% in 2019. During this period, overall antibiotic prescriptions decreased from 1.89 to 1.01 per person-year, with the greatest reductions in macrolides (73.3%) and broad-spectrum antibiotics (57.0%). Antibiotic-treated respiratory tract infections declined from 2.43 to 1.61 episodes per person-year, with the largest decreases in sinusitis (64.7%) and pharyngitis (39.8%).

**Conclusions:**

The findings suggest a temporal association between routine childhood immunization uptake and reduced antibiotic utilization. Although immunization programs are primarily aimed at protecting children from vaccine-preventable diseases, their potential role in complementing antimicrobial stewardship efforts and other factors influencing antibiotic reduction warrants further investigation through more rigorous study designs.

## Introduction

The widespread use and misuse of antibiotics are primary drivers of antimicrobial resistance (AMR), which is a top global public health threat. Vaccination programs, by preventing common diseases for which antibiotics are prescribed, have the potential to reduce antibiotic use and, consequently, the evolutionary pressure toward AMR among bacterial pathogens.^1^ Since bacterial and viral respiratory infections often present with overlapping clinical symptoms, particularly in young children, clinicians face significant challenges in determining appropriate treatment at initial presentation. This diagnostic uncertainty may result in precautionary antibiotic prescribing, especially in pediatric populations where rapid clinical deterioration is a concern. Therefore, the expected impact of vaccination would not necessarily be isolated to vaccines against bacterial diseases—reducing febrile viral infections through vaccination might lead to fewer medical evaluations and opportunities for antibiotic prescribing, as well as reductions in secondary infections that may require antibiotic use.^2^

Few studies have explored contemporaneous trends between widespread vaccination and antibiotic prescribing at the population level, and real-world evidence on these trends remains limited especially among children in the United States (US). A 2023 systematic literature review highlighted this gap, finding moderate certainty from observational studies that influenza and pneumococcal vaccines reduce antibiotic use in children.^3^ Particularly, observational studies on influenza vaccines in children are limited, and research on pneumococcal vaccines has yielded mixed results, with approximately half showing a protective effect and the rest indicating either no effect or a negative impact. Additionally, most studies from the systemic review focused on individual vaccines’ effects on antibiotic use, with few assessing the cumulative impact of multiple routine childhood vaccines. Understanding this cumulative effect is crucial, as routine immunization programs target multiple pathogens simultaneously and may reduce antibiotic use beyond the impact of any single vaccine. Ecological studies offer a valuable approach to investigate this broader impact at population level. Therefore, to address the evidence gap, we analyzed 20 years of data from a large US administrative insurance claims database to provide additional real-world evidence on temporal trends in vaccine uptake, rates of antibiotic prescriptions, and incidence of antibiotic-treated respiratory tract infections in children <5 years of age.

## Methods

### Study design and patient population

This retrospective ecological study used deidentified data from US children with continuous enrollment from birth to at least one year of age in the Merative® MarketScan Commercial Claims and Encounters (CCAE) database between July 1^st^, 2000 and June 30^th^, 2020 (20 epidemiological years from 2000 to 2019, with each year lasting from July 1^st^ of current year to June 30^th^ of next year). The CCAE database covers around 30–40 million people annually, including employees and their dependents, representing approximately 20–30% of the US population with employer-sponsored health insurance.^4,5^ This nationally representative database of commercially insured individuals provides detailed longitudinal medical claims and prescription data, enabling comprehensive trend analyses. Children were required to have full enrollment for the epidemiological year under study and were followed until they no longer contributed data or turned 5 years of age.

Vaccination status was determined using Current Procedural Terminology and National Drug Codes for vaccines offering protection against certain respiratory tract infections, including pneumococcal pneumonia and influenza, which are commonly observed in children, as well as uncommon but highly contagious diseases like *Haemophilus influenzae* type b (Hib) and pertussis. Four vaccine types were selected based on the Centers for Disease Control and Prevention (CDC) recommended immunization schedule for children <5 years of age^6^: 7- or 13-valent pneumococcal conjugate vaccine (PCV7 and PCV13), Hib vaccine, diphtheria-tetanus-acellular pertussis vaccine, and influenza vaccine. The detailed recommendation history for these vaccines is provided in Table 1.^7^ Each year, children were categorized into three groups based on their vaccination status. Those who received at least one dose of all 4 studied vaccines were classified as “received 4 vaccines,” while those who received at least one dose of 1–3 of the studied vaccines were labeled “received 1–3 vaccines”; children with no recorded vaccination codes were considered unvaccinated. The “received 4 vaccines” group represented those with full coverage of the studied vaccines. The “received 1–3 vaccines” group provided an intermediate category allows for evaluating whether partial immunization influenced antibiotic use and infection trends. The unvaccinated group served as a control to measure the effects of not receiving any vaccines. This classification enabled a more detailed analysis of how different levels of immunization relate to antibiotic use.

**Table 1.** Summary of recommendations of vaccines^a^

Group	Vaccine	Vaccination schedule	ACIP recommendation
Pneumococcal Conjugate Vaccines (PCV)	PCV7 (Prevnar, Wyeth/Pfizer)	4-dose series for infants at ages 2, 4, 6, and 12–15 months	PCV7 for children aged ≤23 months and children aged 24–59 months who are at high risk for pneumococcal infection, February 2000 PCV13 for 1) all children aged 2–59 months; and 2) children aged 60–71 months with underlying medical conditions that increase their risk for pneumococcal disease or complications, December 2010
	PCV13 (Prevnar13, Pfizer)	4-dose series at 2, 4, 6 months, and 12–15 months	
Influenza vaccines	LAIV3 (FluMist, MedImmune)	2-dose series at 2–8 years, at least 3 weeks apart	Children 6–23 months old be vaccinated annually against influenza, with implementation scheduled for the fall of 2004, October 2003 Expand influenza recommendations to include vaccination for children 6 months to 18 years old, February 2008 Universal influenza vaccination for those 6 months and older, February 2010 CDC published ACIP’s recommendations for using quadrivalent LAIV (LAIV4) in the 2018–19 influenza season, June 2018
	LAIV4 (FluMist Quadrivalent, MedImmune)	2-dose series at 2–8 years, at least 3 weeks apart	
	ccIIV4 (Flucelvax Quadrivalent, Seqirus)	2-dose series at 2–8 years, at least 3 weeks apart	
*Haemophilus influenzae* type b (Hib) vaccines	Conjugate Hib (PedvaxHIB, MSD)	3-doses series, with first 2 doses 2 months apart for age 2–14 months, and 1 booster dose at age 12–15 months; 1 dose for children aged 15–71 months	Use of any licensed Hib conjugate vaccines for children as young as 15 months old, April 1990 Routine Hib vaccination for infants beginning at 2 months old, January 1991
	Conjugate Hib (ActHIB, Connaught/Mérieux)	4-dose series, with first 3 doses at ages 2, 4, and 6 months, and 1 booster dose at age 15–18 months	
	Hiberix	4-dose series, with first 3 doses at ages 2, 4, and 6 months, and 1 booster dose at age 15–18 months	
Diphtheria-Tetanus-Pertussis (DTaP) vaccine	Infanrix by SmithKline Beecham	5-doses series at 2, 4, 6 and 15–20 months of age, and at 4–6 years of age	DTaP vaccines for all five doses in the vaccination schedule, 1998
	Daptacel by Aventis Pasteur	5-doses series at 2, 4, 6 and 15–20 months of age, and at 4–6 years of age	

ACIP, Advisory Committee on Immunization Practices; CDC, Centers for Disease Control and Prevention; LAIV, live attenuated influenza vaccine.

a Based on the CDC Vaccine History Time line^7^ and CDC ACIP Vaccine-Specific Recommendations.^6^

### Study outcomes

Antibiotic prescriptions for any reason were identified from pharmacy data using National Drug Code identifiers, and grouped into 8 drug classes: penicillins, cephalosporins, macrolides, tetracyclines, quinolones, sulfonamides/trimethoprim, lincosamides, and others. Antibiotic prescriptions were further grouped into broad- and narrow-spectrum based on a previously published classification^8^ (Supplemental Table 1).

Five respiratory tract infections that commonly result in antibiotic prescribing among children^9,10^ in the outpatient setting were assessed using International Classification of Diseases 10th Revision codes from the medical claims data: otitis media, pharyngitis, pneumonia (all-cause invasive and non-invasive pneumonia excluding viral pneumonia), sinusitis, and viral respiratory infection (including viral pneumonia, influenza, viral bronchitis, etc.). Although viral respiratory infections do not require antibiotic use, these conditions were included because prior studies have shown that many viral respiratory infections are empirically treated with antibiotics.^3^

An infection case was considered as an antibiotic-treated case if an antibiotic prescription was observed within 3 days of the start of the case. Antibiotic-treated urinary tract infection (UTI) episodes were used as a benchmark (negative control) to assess trends in antibiotic-treated episodes over time. UTIs were selected because they are primarily caused by pathogens such as *Escherichia coli*, which are not targeted by the routine childhood vaccines examined in this study.^11^ Additionally, UTIs are a common reason for antibiotic prescriptions in young children, making them a stable comparator for evaluating broader trends in antibiotic use that are independent of vaccine-related effects. The rates of antibiotic prescriptions and incidence of antibiotic-treated infection episodes per person-year (PY) were calculated for each epidemiological year.

## Results

### Demographic characteristics

In total, data were available on 6,688,178 children over 20 epidemiological years. The number of children included increased each year from 2000 to 2011 (from approximately 50,000 in 2000 to 564,000 children in 2011), then decreased from 2011 to 2019 (approximately 345,000 in 2019). Across the epidemiological years, the mean age of each cohort ranged from 1.6 to 2.3 years with standard deviations of 1.1 to 1.3 years. While displaying some fluctuation between 2000 and 2009, the age distribution remained relatively stable after 2010, with approximately 46%–53% of children being under 2 years of age. Per year, slightly over half of children were male, and the majority had preferred provider organization/point of service insurance coverage (ranging 63.0% to 74.3%). The proportion of children with high-deductible health plan/consumer-directed health plan insurance coverage increased substantially from 0% in 2000 to 27.9% in 2019, reflecting broader changes in healthcare coverage patterns. Most of the children lived in urban areas, with the proportions ranging from 73.5% to 89.2% across the epidemiological years (Table 2).

**Table 2.** Characteristics of the population of children<5 years of age between 2000 – 2019 with continuous health plan coverage for at least 12 months since birth

	Epidemiological year^a^									
	**2000**	**2001**	**2002**	**2003**	**2004**	**2005**	**2006**	**2007**	**2008**	**2009**
**Number of children, N**	50,476	60,263	83,569	121,619	189,233	226,567	309,105	398,580	462,668	422,528
**Age, mean (SD)**	2.2 ± 1.3	2.2 ± 1.4	1.9 ± 1.3	1.6 ± 1.2	1.6 ± 1.1	1.8 ± 1.1	1.9 ± 1.2	2.0 ± 1.2	2.1 ± 1.2	2.2 ± 1.3
<2 years, %	50.0%	51.2%	61.8%	71.2%	70.1%	63.9%	59.4%	59.4%	52.6%	49.7%
2 – 4 years, %	50.0%	48.8%	38.2%	28.8%	29.9%	36.1%	40.6%	40.6%	47.4%	50.3%
**Male, %**	51.6%	51.6%	51.4%	51.3%	51.2%	51.3%	51.2%	51.2%	51.2%	51.2%
**Region**										
Northeast, %	25.6%	25.1%	19.9%	10.7%	9.7%	11.2%	11.2%	9.8%	8.9%	11.8%
North Central, %	21.6%	22.7%	22.5%	24.6%	22.9%	24.1%	24.9%	25.6%	25.4%	27.6%
South, %	47.1%	44.5%	44.6%	43.5%	42.2%	47.4%	48.4%	50.1%	51.2%	44.7%
West, %	5.6%	7.6%	12.8%	20.7%	23.6%	15.9%	14.3%	13.4%	13.4%	15.6%
Missing/unknown, %	.0%	.1%	.1%	.5%	1.6%	1.4%	1.2%	1.2%	1.1%	.2%
**Urbanicity^b^**										
Urban, %	73.5%	80.6%	80.2%	79.1%	79.8%	84.8%	85.7%	85.5%	86.0%	86.7%
Rural, %	20.1%	19.3%	19.7%	20.5%	18.7%	13.9%	13.2%	13.5%	13.0%	13.2%
Missing, %	6.4%	.1%	.1%	.4%	1.5%	1.3%	1.1%	1.0%	1.0%	.1%
**Health plan types**										
HMO/EPO, %	14.2%	23.1%	24.0%	22.4%	27.0%	23.8%	23.8%	22.7%	21.5%	21.2%
PPO/POS, %	74.3%	68.6%	67.3%	67.8%	63.0%	66.4%	68.4%	69.9%	71.4%	67.8%
HDHP/CDHP, %	.0%	.0%	.0%	.1%	.7%	2.1%	2.5%	3.4%	3.6%	7.4%
FFS, %	11.5%	8.0%	8.2%	6.4%	6.0%	5.7%	4.2%	2.4%	1.8%	1.0%
Missing, %	.0%	.3%	.5%	3.3%	3.3%	2.1%	1.2%	1.5%	1.7%	2.5%
	**2010**	**2011**	**2012**	**2013**	**2014**	**2015**	**2016**	**2017**	**2018**	**2019**
**Number of children, N**	473,556	564,089	527,348	545,069	398,579	407,055	385,281	358,701	355,444	348,448
**Age, mean (SD)**	2.1 ± 1.3	2.1 ± 1.3	2.2 ± 1.3	2.2 ± 1.3	2.3 ± 1.3	2.3 ± 1.3	2.3 ± 1.3	2.3 ± 1.3	2.3 ± 1.3	2.3 ± 1.3
<2 years, %	52.7%	56.2%	51.0%	49.4%	48.0%	46.7%	46.5%	46.2%	47.5%	46.3%
2 – 4 years, %	47.3%	43.8%	49.0%	50.6%	52.0%	53.3%	53.5%	53.8%	52.5%	53.7%
**Male, %**	51.3%	51.5%	51.5%	51.4%	51.4%	51.3%	51.3%	51.3%	51.1%	51.2%
**Region**										
Northeast, %	13.6%	15.4%	16.9%	18.3%	18.3%	16.8%	17.9%	20.1%	20.2%	20.4%
North Central, %	26.9%	25.9%	23.6%	24.0%	22.2%	22.8%	21.6%	23.0%	22.6%	22.9%
South, %	41.8%	38.5%	34.9%	35.2%	44.9%	45.4%	46.4%	42.2%	43.5%	43.8%
West, %	17.1%	18.0%	21.9%	17.3%	13.4%	14.9%	13.9%	14.3%	13.0%	12.6%
Missing/unknown, %	.5%	2.1%	2.8%	5.2%	1.2%	.2%	.2%	.4%	.6%	.2%
**Urbanicity^b^**										
Urban, %	88.0%	86.8%	87.0%	84.2%	89.2%	90.0%	75.9%	74.9%	75.9%	75.2%
Rural, %	11.7%	11.2%	10.3%	10.6%	9.7%	9.9%	9.5%	9.2%	9.1%	9.6%
Missing, %	.4%	2.0%	2.7%	5.1%	1.1%	.1%	14.6%	15.9%	15.0%	15.2%
**Health plan types**										
HMO/EPO, %	21.1%	19.1%	19.5%	13.6%	11.5%	11.1%	12.0%	13.0%	12.5%	12.1%
PPO/POS, %	68.9%	67.8%	65.0%	66.1%	63.9%	63.7%	58.8%	55.2%	55.6%	55.6%
HDHP/CDHP, %	7.3%	9.5%	13.5%	18.7%	22.5%	23.0%	26.1%	27.6%	27.8%	27.9%
FFS, %	.6%	.7%	.7%	.9%	1.2%	1.8%	2.3%	2.7%	2.5%	2.3%
Missing, %	2.0%	2.9%	1.2%	.8%	.9%	.5%	.8%	1.5%	1.6%	2.1%

CDHP, consumer-directed health plan; EPO, exclusive provider organization; FFS, fee-for-service; HDHP, high-deductible health plan; HMO, health maintenance organization; POS, point of service; PPO, preferred provider organization; SD, standard deviation.

a Epidemiological year represents the 12-month period from July 1^st^ of the current year to June 30^th^ of the next year.

b Urbanicity was defined based on metropolitan statistical area in the Merative® MarketScan Commercial Claims and Encounters database.

### Trends in vaccine uptake

**Figure 1.**
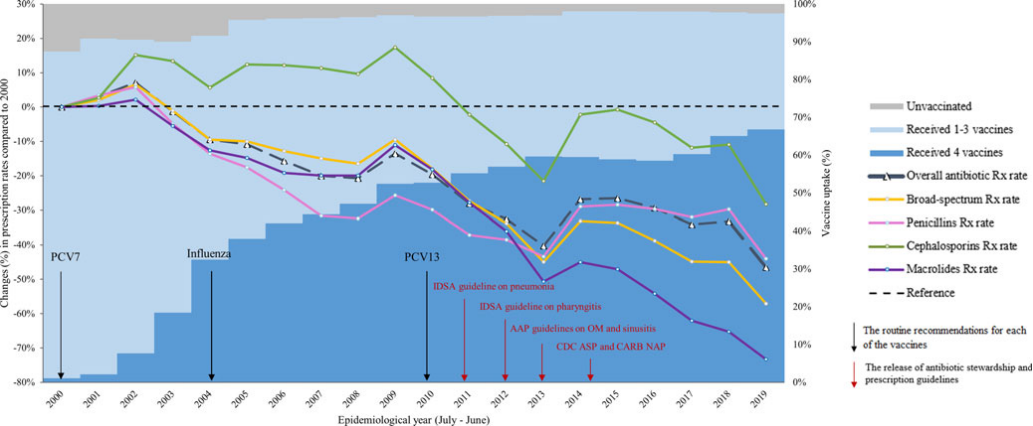
Change in antibiotic prescription rates compared to 2000 (left axis) and vaccine uptake^a^ (right axis) among children <5 years of age by epidemiological year, 2000–2019^b^. AAP, American Academy of Pediatrics; ASP, Antibiotic Stewardship Programs; CARB, Combating Antibiotic-Resistance Bacteria; CDC, Centers for Disease Control and Prevention; IDSA, Infectious Disease Society of America; NAP, National Action Plan; OM, otitis media; Rx, prescription; UTI, urinary tract infection. ^a^ Vaccine uptake was assessed by categorizing children into 3 groups: “received 4 vaccines” if they received ≥1 dose of all 4 vaccine types (pneumococcal, *Haemophilus influenzae* type b, diphtheria-tetanus-pertussis, and influenza); “received 1–3 vaccines” if they received ≥1 dose of 1–3 of the vaccines under study; and “unvaccinated” if they had no vaccination codes for any of the considered vaccines. ^b^ Epidemiological year represents the 12-month period from July 1^st^ of the current year to June 30^th^ of the next year.

During the study period, vaccine uptake in young children increased dramatically, from 32.5% of children receiving at least one dose of the 4 study vaccines in 2004 (the first epidemiological year when all 4 study vaccines were recommended) to 66.8% in 2019. The proportion of unvaccinated children decreased from 8.4% in 2004 to 2.5% in 2019 (Figure 1).

### Trends in antibiotic prescription rates

Antibiotic prescription rates decreased over the study period, from 1.89 prescriptions per PY in 2000 to 1.01 prescriptions per PY in 2019. On aggregate, approximately half of antibiotic prescriptions were broad-spectrum, with proportions being 55.0%–59.0% in 2000–2012, 50.1%–54.5% in 2013–2016, and lower than 50.0% (44.6%–48.1%) in 2016–2019. Penicillins (49.8%–61.4%), cephalosporins (18.5%–25.7%), and macrolides (9.2%–19.0%) were the most commonly prescribed antibiotics (Supplemental Figure 1). During the study period, prescription rates for penicillins decreased from 1.10–1.17 per PY in the early 2000s to .62 per PY in 2019, for cephalosporins from about .40 per PY in the early 2000s to .25 per PY in 2019, and for macrolides from .35 per PY in the early 2000s to .09 per PY in 2019.

Relative to 2000, decreased prescription rates were observed for all antibiotics as a group, as well as for broad-spectrum, penicillin, and macrolide antibiotics. Cephalosporin prescribing was stable during 2000–2009 and decreased during 2009–2019, with some fluctuations in 2014–2018 (Figure 1). The rate of overall antibiotic prescriptions decreased by 46.6% from 2000 to 2019. The greatest rate reduction in 2019 in comparison to 2000 was observed for macrolides (73.3%), followed by broad-spectrum antibiotics (57.0%), penicillins (44.0%), and cephalosporins (28.3%). Decreasing trends on the overall prescription rates were most evident during 2003–2007 (1.3%–20.0%) and 2010–2013 (19.6%–40.3%).

### Trends in incidence of antibiotic-treated respiratory tract infections

The incidence of overall antibiotic-treated infection episodes decreased during the study period, from 2.43 episodes per PY in 2000 to 1.61 episodes per PY in 2019. Otitis media was the most common infection, responsible for 43%–48% of all antibiotic-treated respiratory tract infection episodes, followed by viral respiratory infections (30%–38%), and pharyngitis (11%–15%). Sinusitis and pneumonia were less common, together contributing to <10% of antibiotic-treated respiratory tract infection episodes (Supplemental Figure 2).

**Figure 2.**
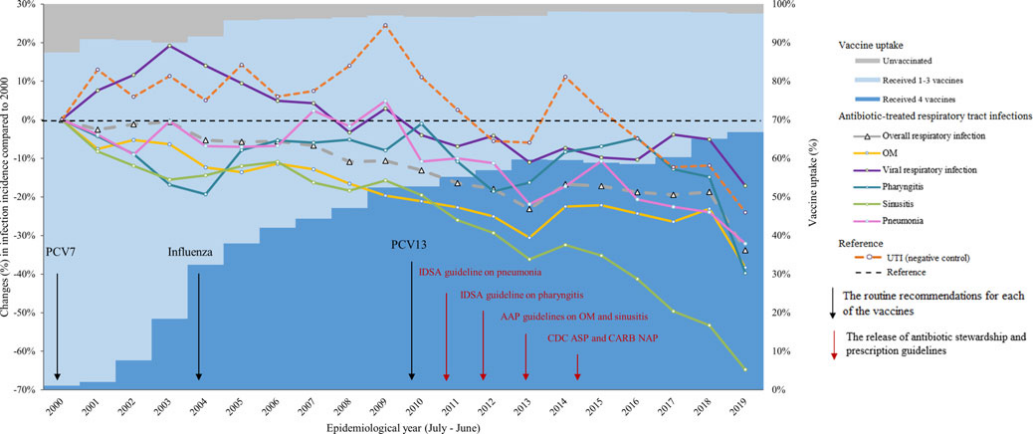
Change in incidence of antibiotic-treated infections compared to 2000 (left axis) and vaccine uptake^a^ (right axis) among children <5 years of age by epidemiological year, 2000–2019^b^. AAP, American Academy of Pediatrics; ASP, Antibiotic Stewardship Programs; CARB, Combating Antibiotic-Resistance Bacteria; CDC, Centers for Disease Control and Prevention; IDSA, Infectious Disease Society of America; NAP, National Action Plan; OM, otitis media; PVC, pneumococcal conjugate vaccines; Rx, prescription; UTI, urinary tract infection. ^a^ Vaccine uptake was assessed by categorizing children into 3 groups: “received 4 vaccines” if they received ≥1 dose of all 4 vaccine types (pneumococcal, *Haemophilus influenzae* type b, diphtheria-tetanus-pertussis, and influenza); “received 1-3 vaccines” if they received ≥1 dose of 1–3 of the vaccines under study; and “unvaccinated” if they had no vaccination codes for any of the considered vaccines. ^b^ Epidemiological year represents the 12-month period from July 1^st^ of the current year to June 30^th^ of the next year.

Compared with 2000, decreasing trends were observed for overall antibiotic-treated respiratory tract infections and for each individual case. The incidence of overall antibiotic-treated respiratory tract infections decreased by 33.8% in 2019 compared to 2000. The greatest rate reduction was observed for sinusitis (64.7%), followed by pharyngitis (39.8%), otitis media (38.3%), pneumonia (32.1%), and viral respiratory infection (17.1%). In contrast, antibiotic-treated UTI incidence remained relatively stable during the study period, with a notable decrease of 4.9%–24.0% during 2016–2019, similar to the 3.8%–17.1% decrease observed for viral respiratory infections (Figure 2).

Despite the absolute number of episodes declining over time, the relative proportions of antibiotic-treated respiratory tract infections remained fairly stable throughout the study period, with otitis media consistently representing the largest share (43%–48%). This pattern suggests that the observed reductions in antibiotic prescribing affected all respiratory conditions proportionally rather than being driven by changes in a single condition.

## Discussion

This study reveals four concurrent trends in young children from 2000 through 2019: an increase in vaccine uptake, a decrease in antibiotic prescriptions, a decline in antibiotic-treated respiratory tract infections, and relatively stable rates for UTIs. Vaccine uptake increased in 2004, following the recommendation for annual influenza vaccination, and it continued to increase in subsequent years. During 2004–2008, when all four of the study vaccines were recommended, rates of most antibiotic prescriptions and all respiratory tract infection episodes decreased. Notable decreases in antibiotic prescriptions and infection episodes during 2010–2013 coincided with the recommendation for use of PCV13 and the recommendation for universal influenza vaccination.

Although causality cannot be inferred due to the ecological study design, the observed trends are consistent with the notion that reducing vaccine-preventable diseases may decrease antibiotic use. Data support this for some vaccines, such as PCV, which reduces the risk of acute otitis media and antibiotic use among the pediatric population, according to a prior meta-analysis.^3^ Influenza vaccines, while aimed at reducing influenza infection, which should not be treated with antibiotics, have also been shown to significantly reduce antibiotic use by lowering acute respiratory infections that often lead to empiric antibiotic use and secondary bacterial infections.^3^

Our findings align with previous studies on the impact of vaccines on antibiotic prescriptions. Klein et al. examined the relationship between influenza vaccine coverage and the number of antibiotic prescriptions per 1,000 residents during 2010–2017.^12^ The study found that in the pediatric population (aged 0–18 years), a 10% increase in influenza vaccine coverage was associated with 6% decrease in antibiotic prescription rates, equivalent to a decrease of 15.2 prescriptions per 1,000 residents. Consistent with our results, the greatest reduction was observed for macrolides, which are commonly prescribed for respiratory tract infections, including those caused by atypical pathogens, particularly in patients with penicillin allergy. Several ecological studies reported similar findings, such as increased influenza vaccine uptake being associated with reduced antibiotic use at the population level, and birth cohorts after the introduction of PCV programs showing a lower risk of antibiotic use.^12–14^ However, these studies either did not focus on young children in early life, when they are more vulnerable and vaccines are crucial for preventing infections and reducing antibiotic use,^12,13^ or did not directly assess vaccine uptake at the population level.^14^ Moreover, these studies often had a limited scope, focusing solely on either the influenza or pneumococcal vaccine. In contrast, this study examines four routine childhood vaccines that protect against bacterial pathogens known to cause respiratory infections requiring antibiotics (pneumococcal pneumonia, *H. influenzae* type b, and pertussis) as well as influenza virus, providing a more comprehensive view of real-world impacts.

During 2000–2019, antibiotic prescription practices shifted significantly, driven by evolving clinical guidelines and intensified efforts to combat AMR. These changes aimed to reduce unnecessary antibiotic use, improve treatment precision, and promote antimicrobial stewardship. From 2011 to 2013, the Infectious Disease Society of American (IDSA) released antibiotic stewardship and updated guidelines for pneumonia and pharyngitis, reinforcing appropriate antibiotic selection and duration.^15,16^ Concurrently, the American Academy of Pediatrics (AAP) issued guidelines for otitis media and sinusitis, emphasizing watchful waiting, stricter diagnostic criteria, and more judicious antibiotic use.^17,18^ In 2014, the CDC launched the Core Elements of Hospital Antibiotic Stewardship Programs to optimize antibiotic use in healthcare settings, followed by the 2015 National Action Plan for Combating Antibiotic-Resistance Bacteria, which strengthened national AMR prevention efforts.^19,20^ These coordinated initiatives likely contributed to the decline in antibiotic prescription observed during the study period.

Before the release of the IDSA and AAP guidelines in 2011, antibiotic prescriptions for respiratory infections had been gradually decreasing, possibly due in part to increased vaccine uptake. Between 2011 and 2017, vaccine uptake plateaued while antibiotic prescriptions for respiratory infections continued to decline significantly, suggesting a stronger influence of stewardship efforts. After 2017, a slight increase in vaccine uptake coincided with a sharper decline in antibiotic prescriptions, indicating a combined effect of vaccination and stewardship initiatives. Notably, antibiotic-treated viral respiratory infections had a substantial decline after the introduction of the influenza vaccine into the routine pediatric vaccination schedule in 2004, even before major stewardship initiatives began in 2011. This highlights the role of influenza vaccination in preventing influenza, the most common viral respiratory infection, and reducing inappropriate antibiotic use. Meanwhile, there were also efforts to curb empiric prescribing for UTI, focusing on proper diagnosis, identification of asymptomatic bacteriuria, and use of narrow-spectrum antibiotics.^21^ Despite a notable decrease in antibiotic-treated UTI in the late 2010s, the overall trend showed little change compared to respiratory infections, especially during 2000–2014. One possible explanation is that rising vaccine uptake during this period may have specifically reduced bacterial respiratory infections, leading to a greater reduction in antibiotic prescribing for respiratory infections than for UTIs. This temporal overlap between increasing vaccine uptake and enhanced stewardship efforts makes it challenging to isolate their individual contributions to reduced antibiotic use. However, the steady decline in antibiotic prescriptions beginning before major stewardship initiatives suggests that vaccination may have played an important early role in this trend.

Beyond vaccination and stewardship initiatives, several factors may have contributed to the decreased antibiotic use. First, the increasing availability of rapid viral diagnostic tests at the point of care has been shown to reduce inappropriate antibiotic use in children by distinguishing viral from bacterial infections.^22,23^ Second, the expansion of managed care among children likely influenced prescribing behavior by discouraging inappropriate antibiotic use through stricter formulary controls and provider oversight.^24,25^ Third, socioeconomic status (SES) impacts antibiotic use through healthcare access, baseline infection risk, and healthcare-seeking behaviors. Lower SES is associated with poor housing conditions, increased exposure to infectious agents, leading to a higher burden of infections, and barriers to healthcare access, including limited diagnostic resources and concerns about follow-up care.^26–28^ Fourth, emerging antimicrobial resistance patterns has affected antibiotic selection. In the 2010s, *S. pneumoniae* showed persistently high resistance rates, particularly to macrolides among U.S. children, prompting a shift in prescribing patterns toward alternative agents.^29^ Consistently, our study found a steeper decline in macrolide prescriptions compared to alternatives like penicillins. Fifth, data suggest that breastfeeding rates in the U.S. increased substantially during the study period;^30^ in as much as breastfeeding is protective against acute otitis media^31^ and other common infections,^32^ increases in breastfeeding could have driven down antibiotic use by reducing the occurrence of acute infectious syndromes that would otherwise have prompted antibiotic therapy. Finally, the increasing use of electronic health records during the study period may have contributed to more judicious antibiotic prescribing through improved documentation of past medical history, automated clinical decision support, and better tracking of prescribing patterns.^33^ These factors, collectively, may have complemented vaccination efforts in shaping the observed trends in antibiotic use.

The observed trends in antibiotic prescribing patterns—particularly the marked decrease in broad-spectrum antibiotic and macrolide use—align with antimicrobial stewardship principles promoting the use of narrow-spectrum agents when appropriate. This suggests that multiple public health initiatives, including both vaccination programs and prescribing guidelines, may have synergistically contributed to more appropriate antibiotic use in young children.

This study has common limitations of retrospective claims databases. It explored the relationship between vaccine uptake and antibiotic use but couldn’t establish causality given the ecological design. A patient-level evaluation, such as a matched cohort study comparing children with varying vaccination statuses, was not optimal *for describing the time-dependent impact associated with dynamic* vaccine uptake over time. The dynamic nature of vaccination coverage, influenced by policy changes, seasonal variations, and healthcare access, further complicates cohort-based analyses. Given these constraints, an ecological approach using a large administrative claims database provided the most viable means to assess population-level trends. This study is subject to confounding biases, including temporal effects and variations in the number and characteristics of the selected population throughout the study period. Additionally, this study included only children covered by commercial health plans, not including the more vulnerable Medicaid and uninsured populations with likely lower vaccine uptake. Furthermore, our categorization of vaccination status (“received 4 vaccines,” “received 1–3 vaccines,” and “unvaccinated”) presents an inherent limitation, as it treats all vaccine combinations within each category as equivalent. This simplified grouping may obscure differential impacts of specific vaccine combinations on antibiotic utilization, as the protective effects against respiratory infections likely vary depending on which specific vaccines a child received.

In conclusion, this ecologic study suggests that vaccination programs may contribute to reduced antibiotic use in young children, reinforcing findings from prior smaller controlled studies. While various factors besides vaccination may also play a role, future prospective cohort studies using longitudinal patient-level data are warranted to validate this association. These findings highlight the potential policy implications of integrating vaccination programs into antimicrobial stewardship efforts to help reduce unnecessary antibiotic use, ultimately supporting public health efforts to combat antibiotic resistance.

## Supporting information

10.1017/ash.2025.10044.sm001 Eiden et al. supplementary material Eiden et al. supplementary material

## Supplementary material

To view supplementary material for this article, please visit https://doi.org/10.1017/ash.2025.10044

## Research transparency and reproducibility

The data that support the findings of this study are available from Merative. Restrictions apply to the availability of these data, which were used under license for this study. Data are available upon request from the authors with the permission of Merative.
