# Supplementary material for: Temporal trends in vaccination and antibiotic use among young children in the United States, 2000–2019
Source: Antimicrob Steward Healthc Epidemiol. 2025 Jul 11;5(1):e151. doi: 10.1017/ash.2025.10044 (PMC12247003; doi:10.1017/ash.2025.10044)
Supplement: Eiden et al. supplementary material [file S2732494X25100442sup001.docx]

Supplemental Material

Supplemental Table 1. List of antibiotics

| **Drug class** | **Overall** | **Broad spectrum** |
| --- | --- | --- |
| **Penicillins** |  |  |
|  | Penicillins |  |
|  | Amoxicillin-clavulanate | Amoxicillin-clavulanate |
|  | Amoxicillin-clarithromycin-lansoprazole | Amoxicillin-clarithromycin-lansoprazole |
| **Cephalosporins** |  |  |
|  | Cephalosporins | Second-generation |
|  | Loracarbef | Cefaclor |
|  |  | Cefotetan |
|  |  | Cefprozil |
|  |  | Cefuroxime |
|  |  | loracarbef |
|  |  | Third-generation |
|  |  | Cefixime |
|  |  | Cefdinir |
|  |  | Cefditoren |
|  |  | Cefpodoxime |
|  |  | Ceftazidime |
|  |  | Ceftibuten |
|  |  | Ceftriaxone |
|  |  | Forth-generation |
|  |  | Fifth-generation |
| **Macrolides** |  |  |
|  | Macrolides | Azithromycin |
|  |  | Clarithromycin |
|  | Telithromycin | Telithromycin |
| **Tetracyclines** |  |  |
|  | Tetracyclines | Lidocaine-oxytetracycline |
| **Quinolones** |  |  |
|  | Fluoroquinolones | Quinolones |
|  | Other quinolones | First-generation |
|  | Cinoxacin | Ciprofloxacin |
|  |  | Norfloxacin |
|  |  | Ofloxacin |
|  |  | Second-generation |
|  |  | Gatifloxacin |
|  |  | Levofloxacin |
|  |  | Moxifloxacin |
|  |  | Trovafloxacin |
|  |  | Third-generation |
|  |  | Gemifloxacin |
|  |  | Cinoxacin |
| **Sulfonamides/trimethoprim** |  |  |
|  | Sulfonamides |  |
|  | Trimethoprim |  |
|  | Sulfonamides-trimethoprim combinations |  |
|  | Trimethoprim-Sulfadiazine |  |
|  | Sulfamethoxazole-Trimethoprim |  |
|  | Trimethoprim/Suflametopyrazine |  |
|  | Trimethoprim-Sulfametrole |  |
|  | Trimethoprim/Sulfamoxole |  |
|  | Tetroxoprim-Sulfadiazine |  |
|  | Tetroxoprim-Sulfadiazine-Bromhexine |  |
|  | Trimethoprim-Sulfamethoxazole-Attapulgite |  |
|  | Trimethoprim-Sulfamethoxazole-Bromhexine |  |
|  | Trimethoprim-Sulfamethoxazole-GG |  |
|  | Trimethoprim-Sulfamethoxazole-Iodinated Glycerol |  |
|  | Trimethoprim-Sulfamethoxazole-Bromhexine-Ethamivan |  |
|  | Trimethoprim-Sulfamethoxazole-GG-Ammon Cl |  |
|  | Trimethoprim-Sulfamethoxazole-Bromhexine-Ethamivan-GG |  |
| **Lincosamides** |  |  |
|  | Lincosamides | Lincosamides (including clindamycin) |
| **Others** |  |  |
| **Aminoglycosides** |  |  |
|  | Aminoglycosides |  |
| **Rifamycins** |  |  |
|  | Rifabutin | Rifabutin |
|  | Rifampin | Rifampin |
|  | Rifapentine | Rifapentine |
|  | Rifaximin | Rifaximin |
| **Polypeptides** |  |  |
|  | Polymyxins |  |
|  | Colistimethate |  |
|  | Bacitracin |  |
|  | Methenamine |  |
|  | Methenamine-sodium acid phosphate |  |
| **Monobactams** |  |  |
|  | Monobactams |  |
| **Carbapenems** |  |  |
|  | Carbapenems | Carbapenems |
|  |  | Meropenem / Meropenem & Sodium Chloride |
|  |  | Imipenem-Cilastatin / Meropenem-Vaborbactam |
|  |  | Imipenem-Cilastatin-Relebactam / Imipenem-Cilastatin and Sodium Chloride |
| **Oxazolidinones** |  |  |
|  | Oxazolidinones |  |
| **Nitrofurans** |  |  |
|  | Furazolidone | Furazolidone |
|  | Nitrofurantoin | Nitrofurantoin |
| **Streptogramins** |  |  |
|  | Quinupristin-Dalfopristin |  |
| **Lipopeptide** |  |  |
|  | Daptomycin |  |
| **Glycopeptides** |  |  |
|  | Glycopeptides |  |
| **Miscellaneous** |  |  |
|  | Fosfomycin | Fosfomycin |
|  | Ethambutol | Ethambutol |
|  | Ethionamide | Ethionamide |
|  | Metronidazole | Metronidazole |
|  | Tinidazole | Tinidazole |
|  | Chloramphenicol | Chloramphenicol |
|  | Isoniazid |  |
|  | Pyrazinamide |  |
| **Anti-infective Combinations** |  |  |
|  | Erythromycin-Sulfisoxazole | Erythromycin-Sulfisoxazole |
|  | Trimethoprim-Rifampin | Trimethoprim-Rifampin |
|  | Metronidazole-Spiramycin | Metronidazole-Spiramycin |
|  | Furazolidone-Kaolin-Pectin-Neomycin-Homatropine | Furazolidone-Kaolin-Pectin-Neomycin-Homatropine |
|  | Furazolid-Colistin-Dicyclomine-Kao-Pectin | Furazolid-Colistin-Dicyclomine-Kao-Pectin |
|  | Furazolidone-Colistin-Iodoquinol-Dicyclomine | Furazolidone-Colistin-Iodoquinol-Dicyclomine |
|  | Chloramphenicol-Colistimethate Sodium w/ Dibucaine | Chloramphenicol-Colistimethate Sodium w/ Dibucaine |
|  | Chloramphen-Colistin-CPM-GG-Dipyrone w/ Dibucaine | Chloramphen-Colistin-CPM-GG-Dipyrone w/ Dibucaine |
|  | Chloramphen-Colistimethate-CPM-GG-Dipyrone w/ Dibucaine | Chloramphen-Colistimethate-CPM-GG-Dipyrone w/ Dibucaine |
|  | Metronidazole Benzoate-Iodoquinol |  |
|  | Metronidazole-Iodoquinol |  |
|  | Metronidazole-Nystatin |  |
|  | Metronidazole-Scopolamine Butylbromide |  |
|  | Furazolidone-Polynoxylin-Dicyclomine | Furazolidone-Polynoxylin-Dicyclomine |
|  | Furazolidone-Pectin-Atta-Homatrop | Furazolidone-Pectin-Atta-Homatrop |
|  | Furazolidone-Pipenzolate Br w/ Pectin-Attapulgite | Furazolidone-Pipenzolate Br w/ Pectin-Attapulgite |
|  | Nifurzide-Pectin | Nifurzide-Pectin |
|  | Nifuroxazide-Kaolin-Pectin | Nifuroxazide-Kaolin-Pectin |
|  | Colistin-Kaolin-Pectin |  |

Supplemental Figure 1. Antibiotic prescription rates among children < 5 years of age by epidemiological year^1^, 2000-2019

a. Antibiotic prescription rates by broad- and narrow-spectrum

b. Antibiotic prescription rates by class

[1] Epidemiological year represents the 12-month period from July 1^st^ of the current year to June 30^th^ of the next year.

Supplemental Figure 2. Antibiotic-treated respiratory tract infection among children < 5 years of age by epidemiological year^1^, 2000-2019

[1] Epidemiological year represents the 12-month period from July 1^st^ of the current year to June 30^th^ of the next year.
